# Takotsubo Cardiomyopathy: A Cardiac Syndrome Mimicking Acute Myocardial Infarction in a Liver Transplant Recipient

**DOI:** 10.4021/cr35e

**Published:** 2011-03-25

**Authors:** Maria M Anders, Pablo D Comignani, Rocio Couce, Nadia Prini, Alina R Zerega, Mariano Santopinto, Gustavo Devetach, Emilio G Quinonez, Nicolas Goldaracena, Lucas McCormack, Ricardo C Mastai

**Affiliations:** a Liver Transplant Program, Hospital Aleman, Buenos Aires, Argentina

**Keywords:** Takotsubo cardiomyopathy, Liver transplantation, Heart failure, Intra-aortic balloon pumping, Myocardial infarction, Left ventricular apical ballooning

## Abstract

Takotsubo cardiomyopathy (TTC) is a rare clinical syndrome defined as a profound but reversible left ventricular dysfunction in the absence of coronary artery disease. We describe the clinical features and management of TC manifesting in the postoperative period in a patient undergoing liver transplantation. Two days after surgery, the patient developed clinical features of acute myocardial infarction. Ecochardiography revealed hypokinesis of the left ventricle. Coronary angiography revealed normal arteries without any stenosis or obstruction. The patient required vasopressor and inotropic support. The placement of intra-aortic balloon pump had a beneficial effect on the management of heart failure. The patient had a complete recovery of cardiac function 40 days after surgery. TC is a possible occurrence after liver transplant. Awareness of this condition is essential as early diagnosis and prompt management can save the patient’s life.

## Introduction

Takotsubo cardiomyopathy (TTC), also known as apical ballooning syndrome or stress cardiomyopathy, is a reversible cardiac syndrome characterized by transient left ventricular dysfunction, electrocardiographic changes that can mimic acute myocardial infarction, and minimal release of myocardial enzymes in the absence of significant coronary artery disease [1]. This syndrome, which was first described in the Japanese population, is frequently precipitated by a stressful event [2].

We describe a case of Takotsubo cardiomyopathy occurring at our institution in a patient following liver transplantation. In addition, the diagnostic criteria and therapeutic modalities in the management of this acute cardiomyopathy are discussed.

## Case Report

**Figure 1:**
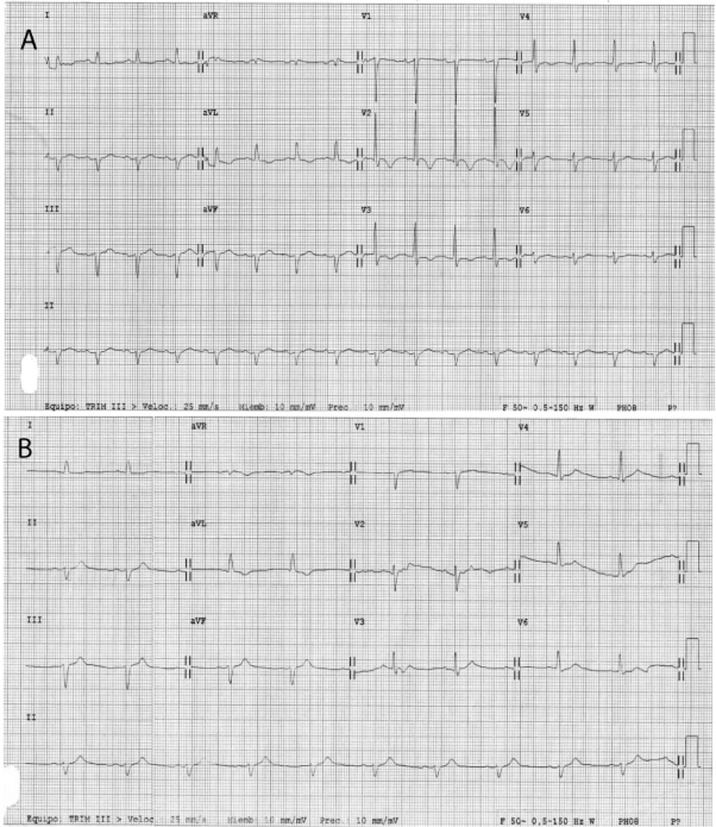
ECG two days after liver transplantation showing ST elevation suggesting extensive anterior wall myocardial ischemia (A) and a normal tracing after recovery (B) in the patient with takotsubo cardiomyopathy.

**Figure 2:**
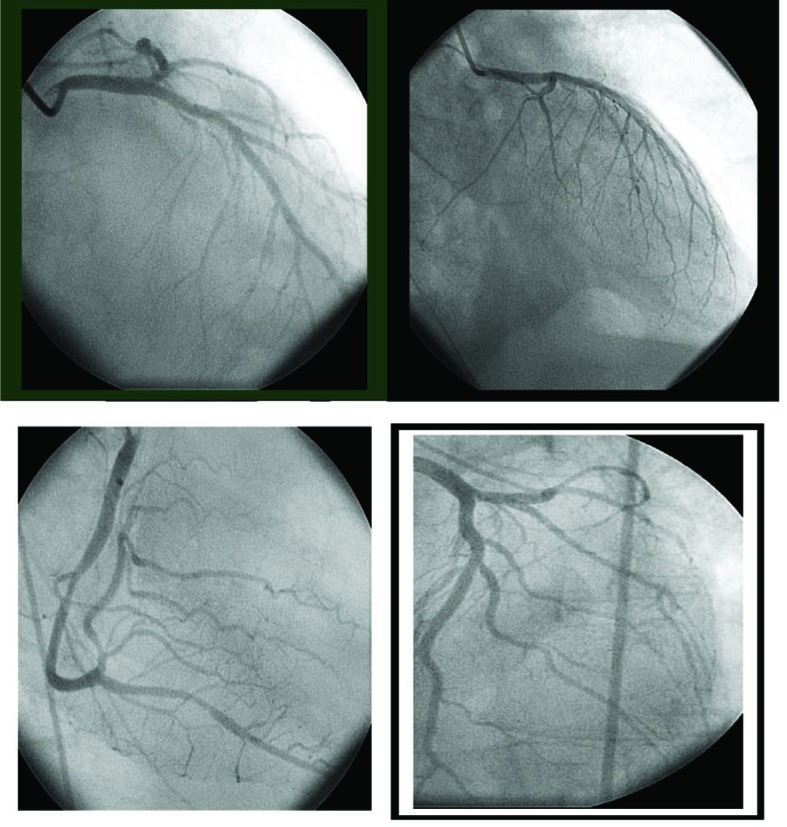
Coronary angiography in the patient with takotsubo cardiomyoptahy. Obstructive coronary artery disease was not seen in the right or left coronary artery.

A 66-year-old man presenting with end-stage liver disease secondary to alcohol consumption and a preoperative Model for End-Stage Liver Disease (MELD) score of 23 was referred to our center for liver transplantation. His past medical history included autologous bone marrow transplantation 16 years ago after a Hodgkin lymphoma. The patient had complications presented as portosystemic encephalopathy and spontaneous bacterial peritonitis. Preoperative medications included spironolactone, lactulose, norfloxacin and rifaximin. Cardiac evaluation before surgery was normal. The pretransplant evaluation included an unremarkable transthoracic echocardiogram with normal left ventricular function with an ejection fraction of 60%. Eight units of fresh frozen plasma were transfused during the surgery in order to improve coagulation time. There was no need to transfuse red cells. The patient did not require pressors during the surgical procedure and had no significant hemodynamic changes. The immediate post transplant period was good until 48 hours after transplant when ST elevation was observed on the telemetry monitor. A 12-lead electrocardiogram showed signs of cardiac ischemia (Fig. 1). Peak total creatine kinase levels were 522 U/L (normal 0 - 25) and troponin levels were 0.55 ng/ml (normal 0 - 0.01). Right heart catheterization showed cardiogenic shock with a cardiac index of 1.7 l/min/m^2^. An emergent echocardiographic evaluation revealed an akinesia of the apical segment of the left ventricle and normal contraction of basal segments of the left ventricle, showing a typical aspect of end-systolic left ventricular ballooning. Estimated left ejection fraction was approximately 28%. Coronary angiography showed the absence of obstructive coronary artery disease (Fig. 2). A preliminary diagnosis of Takotsubo cardiomyopathy was made. Treatment included vasodilators (nitroglycerine and hidralazine) and beta blockers. The patient recovered from this event; however, he developed increasing renal failure and encephalopathy. During the next days, a right-sided pleural effusion gradually increased. The patient became hypoxic and experienced acute respiratory failure, requiring emergent orotracheal intubation and pleuroscentesis (placement of pigtail catheter). At this time, the patient was hemodynamically unstable and on high doses of norepinephrine and dobutamine. He also required amiodarone to revert an acute atrial fibrillation. A new echocardiogram estimated a left ejection fraction of 9%. An intra-aortic balloon pump with 1 : 1 support allowed tapering of pharmacologic treatment. Cardiac index and wedged pulmonary pressure values were 1.7 ml/min/m^2^ and 23 mmHg, respectively. On postoperative day 35, inotropic and vasotropic dependence was significantly decreased, and echocardiography revealed an ejection fraction of 30%. By postoperative 23 day, in the light of improving hemodynamics, it was decided remove the intra-aortic balloon pump. By the following days, the patient was completely weaned from all inotropic, vasotropic, mechanical, respiratory and renal support. The patient’s subsequent hospital course was unremarkable and he was discharged 40 days after liver transplantation in good condition, with no signs of cardiac insufficiency and a well-functioning liver graft. Outpatient transthoracic echocardiogram 4 weeks later showed normal left ventricular function with no wall motion abnormalities, and electrocardiogram showed normal findings with resolution of ST segment elevation (Fig. 1).

## Discussion

During the last years, there have been several reports of patient with profound, reversible left ventricular dysfunction after sudden emotional stress [1-3]. This syndrome was described by Japanese authors in 1990, and referred to as “takotsubo cardiomyopathy” [2]. More recently, the term “transient left ventricular ballooning” has been used to describe similar cardiac contractile abnormalities in patients after emotional or physical stress [3]. The prevalence of the disease is unknown. However, since TTC has become more widely recognized and more specific criteria have been established, higher prevalence rates have been reported [4].

In our patient the diagnosis of TTC was based on: 1) a reversible wall motion abnormality; 2) transient ST-T segment abnormalities on the ECG; 3) minimal evidence of coronary artery stenosis, vasospasm and disturbance of microcirculation; and 4) physical or emotional stress as triggering factor. In liver transplant patients, high levels of stress can be expected, and in the case of a postoperative complication, this can be significantly increased. In fact, this syndrome has been recently reported in the perioperative setting of liver transplantation [5-7].

Despite an increased awareness of TTC, the pathophysiologic mechanism remains unknown. Leading hypotheses describe several etiologies including impaired myocardial perfusion, cardiac myocyte injury and metabolic dysfunction induced by physical or emotional stress [2, 8, 9]. Of these, catecholamine-mediated myocardial injury remains the most favored explanation [10]. Recently, both clinical and experimental studies, demonstrate this last finding. In this regard, a marked increase in catecholamine levels was observed in patients with stress-induced TTC [11]. Moreover, animal studies show that a prior treatment with α and β antagonists prevents sympathetic-induced TTC [12]. On the other hand, microvascular dysfunction and spasm have also been thought to be the cause of TTC. A decrease in coronary flow reserve, measured by different methods, was observed in patients with TTC [13-15]. However, at present it remains unknown whether microsvascular dysfunction plays a causative role in TTC or whether, when observed, it is a consequence of a primary myocardial impairment process.

As with the mechanisms that produce this syndrome, the optimal treatment for the TTC remains unknown. In our case the management was that of a patient with myocardial ischemia. Like any patient presenting acutely with chest pain, ECG changes and left wall motion abnormalities underwent a coronary angiography, where minimal changes were observed. After having suspected the diagnosis of TTC, a close monitoring for the development of cardiogenic shock or malignant arrhytmias was indicated. In this regard, it was necessary to use amiodarone to reverse an acute atrial fibrillation. Also, due to pump failure unresponsive to standard drug treatments an intra-aortic balloon counterpulsation was indicated. In this case the mechanical device was able to normalize the hemodynamic changes and made possible the discharge of the patient from the hospital after device removal.

In conclusion, takotsubo or stress cardiomyopathy is an increasingly type of acquired cardiomyopathy in patients undergoing liver transplantation. The clinical picture closely resembles that of an acute coronary syndrome. Prognosis is good with appropiate supportive management in the acute phases. Finally, as ocurred in our patient, the use of percutaneous intra aortic (ventricular) assist device was successful in the treatment of severe cardiogenic shock associated with TTC.

## References

[1] Brenner ZR, Powers J (2008). Takotsubo cardiomyopathy. Heart Lung.

[2] Dote K, Sato H, Tateishi H, Uchida T, Ishihara M (1991). [Myocardial stunning due to simultaneous multivessel coronary spasms: a review of 5 cases]. J Cardiol.

[3] Bybee KA, Kara T, Prasad A, Lerman A, Barsness GW, Wright RS, Rihal CS (2004). Systematic review: transient left ventricular apical ballooning: a syndrome that mimics ST-segment elevation myocardial infarction. Ann Intern Med.

[4] Facciorusso A, Vigna C, Amico C, Lanna P, Troiano G, Stanislao M, Valle G (2009). Prevalence of Tako-Tsubo Syndrome among patients with suspicion of acute coronary syndrome referred to our centre. Int J Cardiol.

[5] Tiwari AK, D'Attellis N (2008). Intraoperative left ventricular apical ballooning: transient Takotsubo cardiomyopathy during orthotopic liver transplantation. J Cardiothorac Vasc Anesth.

[6] Ramsay MA, Takaoka F, Brown MR, Rosenlof P (1989). Coronary artery vasospasm following placement of a cold liver graft during orthotopic liver transplantation. Anesth Analg.

[7] Lee HR, Hurst RT, Vargas HE (2007). Transient left ventricular apical ballooning syndrome (Takotsubo cardiomyopathy) following orthotopic liver transplantation. Liver Transpl.

[8] Bybee KA, Murphy J, Prasad A, Wright RS, Lerman A, Rihal CS, Chareonthaitawee P (2006). Acute impairment of regional myocardial glucose uptake in the apical ballooning (takotsubo) syndrome. J Nucl Cardiol.

[9] Kurisu S, Inoue I, Kawagoe T, Ishihara M, Shimatani Y, Nishioka K, Umemura T (2003). Myocardial perfusion and fatty acid metabolism in patients with tako-tsubo-like left ventricular dysfunction. J Am Coll Cardiol.

[10] Prasad A, Lerman A, Rihal CS (2008). Apical ballooning syndrome (Tako-Tsubo or stress cardiomyopathy): a mimic of acute myocardial infarction. Am Heart J.

[11] Wittstein IS, Thiemann DR, Lima JA, Baughman KL, Schulman SP, Gerstenblith G, Wu KC (2005). Neurohumoral features of myocardial stunning due to sudden emotional stress. N Engl J Med.

[12] Ueyama T, Kasamatsu K, Hano T, Yamamoto K, Tsuruo Y, Nishio I (2002). Emotional stress induces transient left ventricular hypocontraction in the rat via activation of cardiac adrenoceptors: a possible animal model of 'tako-tsubo' cardiomyopathy. Circ J.

[13] Elesber A, Lerman A, Bybee KA, Murphy JG, Barsness G, Singh M, Rihal CS (2006). Myocardial perfusion in apical ballooning syndrome correlate of myocardial injury. Am Heart J.

[14] Sadamatsu K, Tashiro H, Maehira N, Yamamoto K (2000). Coronary microvascular abnormality in the reversible systolic dysfunction observed after noncardiac disease. Jpn Circ J.

[15] Yoshida T, Hibino T, Kako N, Murai S, Oguri M, Kato K, Yajima K (2007). A pathophysiologic study of tako-tsubo cardiomyopathy with F-18 fluorodeoxyglucose positron emission tomography. Eur Heart J.

